# Complete Genome Sequences of Bacillus cereus Group Phages AaronPhadgers, ALPS, Beyonphe, Bubs, KamFam, OmnioDeoPrimus, Phireball, PPIsBest, YungSlug, and Zainny

**DOI:** 10.1128/MRA.00300-21

**Published:** 2021-08-19

**Authors:** Nora Kostyk, Oluchi Chigbu, Erin Cochran, Jackson Davis, Jacob Essig, Linda Do, Nashwan Farooque, Zainab Gbadamosi, Arathi Gnanodayan, Andrew Hale, Nasita Islam, Ahmed Ismail, Andreus Jordan, Krishna Karamsetty, Perry Tanner, Rahul Warrier, Hasib Zaman, Allison A. Johnson

**Affiliations:** a Center for Biological Data Science, Virginia Commonwealth University, Richmond, Virginia, USA; KU Leuven

## Abstract

Here, we report genome sequences of 10 Bacillus cereus group-infecting bacteriophages. Each virus was isolated from an environmental sample, contained a double-stranded DNA genome, and belonged to the *Myoviridae* family. Nine phages exhibit a conserved genome structure, and one phage appears novel in genome structure, sequence, and protein content.

## ANNOUNCEMENT

Virginia Commonwealth University (VCU) students discovered, characterized, and annotated the genomes of 10 novel bacteriophages, as part of the HHMI SEA-PHAGES program (1). Three host bacteria were used, namely, Bacillus thuringiensis DSM 350, B. thuringiensis serovar kurstaki ATCC 33679, and an environmental isolate newly obtained from the James River and determined by 16S rRNA sequencing to be a strain of Bacillus cereus. Bacillus bacteria are ideally used as phage hosts, as they are ubiquitous in the environment and are able to grow on simple media for teaching.

Environmental samples were collected from soil and water across eastern and central Virginia (Table 1). Phages were discovered through enrichment of environmental sample extracts with host bacteria. Phage samples were purified through multiple rounds of plaque picking, serial dilution, infection of fresh bacterial cultures, and plating for isolated plaques (https://seaphagesphagediscoveryguide.helpdocsonline.com/home). Phage genomic DNA was purified from a high titer lysate using the Promega Wizard kit (Madison, WI). Sequencing libraries were prepared from genomic DNA using the NEBNext Ultra II FS kit (New England BioLabs [NEB], Ipswich, MA) for Illumina MiSeq sequencing (University of Pittsburgh Bacteriophage Institute, 9 genomes) or the Swift 2S Turbo flexible library kit (Swift Biosciences, Ann Arbor, MI) for Illumina HiSeq sequencing (VCU Nucleic Acids Sequencing Facility, Beyonphe). For each genome, a subset of 50,000 150-bp reads were assembled at ∼46× genome coverage using Newbler v2.9. Assemblies yielded single contigs with ends evident by many reads ending at the same position; genome completeness was assessed, and long terminal repeat (LTR) ends for 9 samples were identified by a region of double read depth, using Consed v2.9 (2). YungSlug assemblies showed defined ends without evident long terminal repeats. Phage genes were predicted by GeneMark v4.3 (3), Glimmer v3.02 (4), and ARAGORN v1.2.41 (5), using DNA Master v5.23.5 (http://cobamide2.bio.pitt.edu/computer.htm). Students curated each gene prediction (6) and determined potential functions using HHPred (7) and BLASTP (8). Average nucleotide identity (ANI) values were from DNA Master. All tools were run with default parameters.

**TABLE 1:** Genome characteristics for *Bacillus* phages

Phage name	Bacterial host	Genome length (bp)	No. of tRNAs	Long terminal repeat (bp)	No. of ORFs^*a*^	GC content (%)	GenBank accession no.	Sample type	Sample collection location	No. of reads
AaronPhadgers	B. thuringiensis DSM 350	161,772	3	2,623	304	38.7	MF288919	Soil	Richmond, VA; 37.549497N, 77.451558W	600,467
ALPS	B. thuringiensis serovar kurstaki	161,842	0	2,627	295	38.7	MN038179	Soil	Glen Allen, VA; 37.655944N, 77.56907W	730,477
Beyonphe	B. cereus isolate from James River	163,540	0	2,154	300	37.7	MN038178	James River water	Richmond, VA; 37.5271N, 77.4565W	1,459,694
Bubs	B. thuringiensis DSM 350	162,449	0	2,736	302	38.8	MF288918	Soil	Richmond, VA; 37.544945N, 77.454025W	705,223
KamFam	B. thuringiensis serovar kurstaki	161,830	7	2,426	300	37.9	MH638310	Soil	Chesapeake, VA; 36.696529N, 76.243622W	825,445
OmnioDeoPrimus	B. thuringiensis DSM 350	161,833	0	2,871	294	38.8	MH638311	Soil	Richmond, VA; 37.5487N, 77.4511W	608,048
Phireball	B. thuringiensis serovar kurstaki	162,042	0	2,592	293	38.7	MN038176	Soil	Richmond, VA; 37.6305N, 77.5748W	403,908
PPIsBest	B. thuringiensis serovar kurstaki	162,281	0	2,584	301	38.6	MF288917	Soil	Richmond, VA; 37.5467N, 77.4506W	762,343
YungSlug	B. thuringiensis serovar kurstaki	150,033	0	NA^*b*^	227	37.6	MT416612	James River water	Hopewell, VA; 37.324N, 77.2704W	272,380
Zainny	B. thuringiensis serovar kurstaki	162,692	0	2,661	303	38.7	MF288920	Soil	Chester, VA; 37.309887N, 77.426949W	644,142

a ORFs, open reading frames.

b NA, not applicable.

All 10 phages were determined to belong to *Myoviridae* through transmission electron microscopy of phage particles and/or presence of tail tube and tail sheath genes. They contain double-stranded DNA genomes ranging from 150,033 to 163,540 bp long, with GC contents of ∼38%. LTRs ranged from 2,154 to 2,871 bp. Phages Bubs, OmnioDeoPrimus, Phireball, ALPS, Zainny, PPIsBest, and AaronPhadgers exhibit ANI values from 88% to 97%, while KamFam and Beyonphe exhibit ANIs of 60% to 66%. Those nine genomes exhibit conserved synteny and genome structure. YungSlug appears unique (53% ANI) and deviates by genome structure and synteny.

Our nine longer genomes contain 295 to 304 protein-coding genes. Two genomes contain tRNA genes. Functions were predicted for a range of 13% to 19% of genes, and almost all of the genes matched phage homologs in GenBank. In contrast, the YungSlug genome is ∼10,000 bp shorter and contains 227 protein-coding genes, with 101 of those sequences revealing a BLASTP match of bacterial origin. Furthermore, function was predicted for 36% of its proteins, and 43 proteins have homologs in phages SP-10 and SPO1, derived from a B. subtilis host (9, 10). It will be interesting to look deeply at YungSlug as an atypical B. cereus group phage.

### Data availability.

For each of the following phages, their genomes have been deposited into GenBank under accession numbers: AaronPhadgers, MF288919; ALPS, MN038179; Beyonphe, MN038178; Bubs, MF288918; KamFam, MH638310; OmnioDeoPrimus, MH638311; Phireball, MN038176; PPIsBest, MF288917; YungSlug, MT416612; and Zainny, MF288920. Sequence reads are available under the SRA accession number PRJNA732421.

## References

[1] Jordan TC, Burnett SH, Carson S, Caruso SM, Clase K, DeJong RJ, Dennehy JJ, Denver DR, Dunbar D, Elgin SCR, Findley AM, Gissendanner CR, Golebiewska UP, Guild N, Hartzog GA, Grillo WH, Hollowell GP, Hughes LE, Johnson A, King RA, Lewis LO, Li W, Rosenzweig F, Rubin MR, Saha MS, Sandoz J, Shaffer CD, Taylor B, Temple L, Vazquez E, Ware VC, Barker LP, Bradley KW, Jacobs-Sera D, Pope WH, Russell DA, Cresawn SG, Lopatto D, Bailey CP, Hatfull GF. 2014. A broadly implementable research course in phage discovery and genomics for first-year undergraduate students. mBio 5:e01051-13. doi:10.1128/mBio.01051-13. 24496795 PMC3950523

[2] Russell DA. 2018. Sequencing, assembling, and finishing complete bacteriophage genomes. Methods Mol Biol 1681:109–125. doi:10.1007/978-1-4939-7343-9_9. 29134591

[3] Besemer J, Borodovsky M. 2005. GeneMark: Web software for gene finding in prokaryotes, eukaryotes and viruses. Nucleic Acids Res 33:W451–W454. doi:10.1093/nar/gki487. 15980510 PMC1160247

[4] Salzberg SL, Delcher AL, Kasif S, White O. 1998. Microbial gene identification using interpolated Markov models. Nucleic Acids Res 26:544–548. doi:10.1093/nar/26.2.544. 9421513 PMC147303

[5] Laslett D, Canback B. 2004. ARAGORN, a program to detect tRNA genes and tmRNA genes in nucleotide sequences. Nucleic Acids Res 32:11–16. doi:10.1093/nar/gkh152. 14704338 PMC373265

[6] Pope WH, Jacobs-Sera D. 2018. Annotation of bacteriophage genome sequences using DNA Master: an overview. Methods Mol Biol 1681:217–229. doi:10.1007/978-1-4939-7343-9_16. 29134598

[7] Söding J, Biegert A, Lupas AN. 2005. The HHpred interactive server for protein homology detection and structure prediction. Nucleic Acids Res 33:W244–W248. doi:10.1093/nar/gki408. 15980461 PMC1160169

[8] Altschul SF, Madden TL, Schäffer AA, Zhang J, Zhang Z, Miller W, Lipman DJ. 1997. Gapped BLAST and PSI-BLAST: a new generation of protein database search programs. Nucleic Acids Res 25:3389–3402. doi:10.1093/nar/25.17.3389. 9254694 PMC146917

[9] Yee LM, Matsumoto T, Yano K, Matsuoka S, Sadaie Y, Yoshikawa H, Asai K. 2011. The genome of Bacillus subtilis phage SP10: a comparative analysis with phage SPO1. Biosci Biotechnol Biochem 75:944–952. doi:10.1271/bbb.100921. 21597187

[10] Stewart CR, Casjens SR, Cresawn SG, Houtz JM, Smith AL, Ford ME, Peebles CL, Hatfull GF, Hendrix RW, Huang WM, Pedulla ML. 2009. The genome of *Bacillus subtilis* bacteriophage SPO1. J Mol Biol 388:48–70. doi:10.1016/j.jmb.2009.03.009. 19285085 PMC2666789

